# Application of Echocardiography in Anaesthesia: From Preoperative Risk Assessment to Postoperative Care

**DOI:** 10.7759/cureus.69559

**Published:** 2024-09-16

**Authors:** Kaustuv Das, Jayashree Sen, Aishwarya S Borode

**Affiliations:** 1 Department of Anaesthesiology, Datta Meghe Institute of Higher Education and Research, Wardha, IND

**Keywords:** anesthesia, anesthesiology, cardiology, cardiovascular, echocardiography

## Abstract

Echocardiography has carved out a fundamental niche in anaesthesiology, revolutionizing the monitoring and management of cardiac function during surgery. Clinical practice has changed from simple 2D and 3D echocardiography to more sophisticated applications such as incorporating artificial intelligence. Echocardiography provides detailed real-time information about cardiac anatomy and function, helping anaesthesiologists make better decisions regarding tailoring anesthetic interventions and optimizing patient outcomes. From optimizing hemodynamic management in patients with severe aortic stenosis to fine-tuning fluid and vasopressor therapy in patients with right heart dysfunction, echocardiography has improved the care provided in the perioperative period. These applications permit the demonstration of not only technical advantages that could accrue from echocardiography but are also a part of individualized care to improve the outcomes of patients. The challenges in integrating echocardiography with anaesthesia include operator dependency, a steep learning curve in acquiring echocardiographic skills, and limitations due to patient factors and technological limitations, which lead to poor echocardiographic performance. Additionally, transoesophageal echocardiography (TEE) is an invasive procedure with several potential risks that must be considered cautiously. Continuing education, certification recommendations, and skill development are prerequisites for this echocardiography tool to remain robust and reliable in anaesthesiology. Technological innovation, especially in improving 3D imaging and integration with artificial intelligence, is where a very bright future lies ahead for echocardiography. It would further accelerate the process of echocardiographic evaluation and improve diagnostic accuracy. All these would turn out to be more person-centered for each patient. Anaesthesiologists must, therefore, pace themselves with such developments so these can be appropriately applied in the clinics. In summary, echocardiography became so integrally ingrained into anaesthesia that it propelled the specialty with essential tools anaesthesiologists use to manage patients for optimum outcomes. Its application has difficulties and limitations, but continued professional development and development of echocardiographic technology will make sure that its benefits are maximized. Quickly, echocardiography is becoming central to anaesthesiology's role in optimizing patient care and surgical success as we move into the application of evermore sophisticated echocardiographic techniques.

## Introduction and background

Echocardiography is a cardinal diagnostic modality both in cardiology and anaesthesia. The test applies the principles of ultrasound waves in yielding pictures of the heart. Since it is a non-invasive technique, it is beneficial for giving details of cardiac structure and functions that would help diagnose and manage many conditions related to cardiovascular disease. It has significantly improved the care of patients with cardiovascular diseases in the perioperative phase. This modality has provided real-time visualization of structures and functions of the heart, hence invaluable insight into influencing clinical decision-making and improving patient outcomes [1]. The depth of assessment that echocardiography can undertake regarding cardiac function before surgery will help evaluate ventricular function, wall motion abnormalities, and valvular pathologies, which are essential parameters for cardiac risk stratification. It allows for appropriate adjustment of anesthetic plans for high-risk patients and the institution of measures aimed at lessening possible complications, thus optimizing safety. It will become paramount in the preoperative assessment of patients scheduled to undergo major surgeries wherein undiagnosed cardiac conditions could result in significant morbidity or even perioperative mortality [2]. Bedside echocardiography is essential in continuously assessing a critically ill patient whose clinical status is rapidly changing.

Continuous assessment is very important for a critically ill patient whose clinical status changes rapidly. On the other hand, bedside echocardiography avoids wrong or outdated information management, improving the quality of care. It has also fostered a better understanding of the pathophysiological alterations in the perioperative period. It has developed more refined, targeted anesthetic techniques that optimise overall management for patients with complex cardiovascular diseases. Moreover, the broadened availability of echocardiographic facilities and improved portability of devices extend its use even outside specialized centers into more general clinical practice. Although echocardiography has many advantages, this technique has problems [3]. It requires intensive expertise and training to interpret the images correctly. For example, patient factors such as obesity and lung disease may impact the image quality.

Moreover, transoesophageal echocardiography (TEE) is an invasive test and thus not without risks, although the benefits often outweigh the risks in high-risk surgeries. This would mean immense support through continuous training courses and recertification programs to maintain competence in echocardiographic techniques for anaesthesiologists [4]. To put it the other way around, echocardiography has been brought into the limelight as one of the pillars of anaesthesia for minute and dynamic cardiac assessment in optimizing perioperative care. It improves patient outcomes regarding better preoperative evaluation, intraoperative monitoring, and postoperative management, thus forming one of the key tools of modern anesthetic practice. Improving technology will surely make its role in anaesthesiology integral to enhancing the standard of care delivered to patients afflicted with cardiovascular diseases.

## Review

Overview of different types of echocardiography

Echocardiography modalities have been core instruments of modern cardiology and anaesthesiology. All indications, benefits, and limitations of the test should be considered in context to create appropriateness for the clinical scenario. Some include transthoracic echocardiography (TTE), TEE, stress echocardiography, and intravascular ultrasound (IVUS). The echocardiographic technique applied depends on the clinical question in question, the state of the patient, and current expertise. Technology advances will further enhance such techniques and increase their application for diagnosis and therapy. Knowing the details regarding each type of echocardiography allows the clinician to harness this powerful tool to its best potential and, finally, towards improving patient care and outcomes [5].

TTE is the most common form of echocardiography. It involves putting a transducer on the patient's chest, which sends or transmits ultrasound waves through the chest wall. These bounce back off cardiac structures and return to the transducer, producing moving images of the heart. TTE is used comprehensively as the screening test for heart function and structure. It estimates ventricular function and wall motility abnormalities, the presence of valvular diseases such as stenosis or regurgitation establishes a diagnosis of pericardial effusion, measures cardiac chamber size and wall thickness, and points out congenital heart defects. It is non-invasive, requires no special preparation or sedation, and is readily available in most clinical settings. TTE applications extend to most cardiac evaluations, where vital diagnostic information is derived without causing discomfort to the patient. Obese conditions, lung disease, and chests with wall deformities are some conditions associated with poor image quality on TTE. The major limitation of this examination is that dependency on the operator is pretty significant, and much of the accuracy in interpretation lies in the skill and experience of the operator [6]. Using a specially designed probe introduced into the oesophagus near the patient's heart, imaging of the cardiac structures, particularly at the posterior position that cannot be imaged easily with TTE can be done with precision and remarkable detail. It is mainly useful for TEE in intraoperative monitoring of cardiac surgery, assessment of prosthetic heart valves, diagnosis of endocarditis and vegetation detection, evaluation of complicated congenital heart disease, and guiding catheter-based interventions.

Image qualities obtained with TEE are of better quality, especially the posterior cardiac structures, than those obtained with TTE. Real-time monitoring of cardiac surgeries and interventions also indicates the latter. This approach eludes the pitfalls such as the chest wall and lung tissue; hence, the image is clear.

TEE is an invasive procedure and requires sedation. It has some possible complications, much like oesophagal injury or perforation. It is less available and needs special equipment and trained people. The procedure is uncomfortable and contraindicated in some conditions of the oesophagus [7]. Stress echocardiography is an imaging method that applies exercise or pharmacological stress to the patient, demonstrating how the heart works under stress. The heart has to be imaged before and during the test to outline areas with reduced blood flow. It would commonly be utilized in diagnosing coronary artery disease by stress echocardiography, assessment of the degree of ischemia, functional evaluation of the heart following a heart attack, and the efficacy of treatments for heart disease or its intervention. These techniques can be broadly divided into two major subtypes: exercise stress, whereby the patient exercises on a treadmill or bicycle, and pharmacological stress, whereby various medications such as dobutamine or adenosine are given to the patient to mimic the effect of exercise on the body.

With exercise stress, stress echocardiography is a non-invasive type of functional assessment that delivers information regarding cardiac function during conditions of stress that are not available with resting echocardiography. This dynamic approach allows the identification of ischemia and other stress-induced cardiac abnormalities. Exercise limitation makes it difficult to apply in non-exercisable patients, and the pharmacologic stress is associated with side effects and is relatively contraindicated in some patients. As with every other echo technique, it requires a well-experienced operator in its performance and interpretation [8].

IVUS means introducing a small ultrasound probe through the coronary arteries with a catheter. Mild wall structures can clearly be outlined and assessed concerning coronary artery disease. Overall, IVUS has a role in interventional cardiology concerning the severity assessment of coronary artery stenosis, guidance and optimization of percutaneous coronary interventions, evaluation of results post-stent placement, and follow-ups for restenosis or progression of coronary artery disease. IVUS has brought some accuracy to coronary interventions by providing detailed and high-resolution images of the coronary artery walls and lesions. Real-time feedback is provided, allowing instant evaluation during procedures that may be critical to optimizing patient outcomes. It is an invasive technique as it requires catheterization, and mechanical injury to a vessel cannot be excluded. Thus, it cannot be used as a cardiac assessment screening device in general. It is limited to coronary arteries alone. The procedure requires special machinery, and its operators should be trained as it mandates expertise [9].

3D echocardiography provides considerably longer views of cardiac anatomy than 2D imaging. Other specific utilities of the modality are complex congenital heart diseases, structural heart interventions, and valvular morphology and function assessment [10]. Contrast echocardiography improves imaging in cardiac structures and blood flow by ultrasound contrast agents. It may be used to visualise the endocardial border definition better or identify intracardiac shunts and perfusion within the myocardium [11].

Role of echocardiography in preoperative assessment, intraoperative, and postoperative management

Echocardiography has been a standard of care in surgical patients. It provides information in the assessment before surgery, monitoring during surgery, and management after surgery. This modality imaging gives detailed, real-time images of heart structure and function, thus offering the anaesthesiologist appropriate decisions to promote better outcomes, treatment optimization, and safety.

Preoperative Assessment

Echocardiography plays a quintessential role in assessing preoperative risk and formulating perioperative plans due to its information on cardiac structure and detailed cardiac function. It can establish undiagnosed cardiac conditions, evaluate the ventricular function, and alter anaesthesia planning.

Detection of Cardiac Conditions: Echocardiography also identifies structural abnormalities not apparent through other diagnostic methods, including hypertrophic cardiomyopathy, valvular diseases, and structural anomalies. The early identification of these conditions is critical because it has the most significant impact on perioperative risks and anaesthetic and surgical interventions [12].

Ventricular Function: Other parameters, such as ejection fraction (EF) and diastolic function, also offer essential information regarding myocardial performance. A low EF is very suggestive of low cardiac function and an increased postoperative risk. A precise evaluation of the status of ventricular function determines the extent to which patients can withstand surgical stress [13].

Influence on Anaesthesia Planning: Echocardiography helps in planning for heart valve surgeries and major cardiovascular interventions. In detail, it provides information about the degree of valvular stenosis or regurgitation and thus guides decisions on valve repair or replacement and the appropriate anaesthetic approach [14].

Intraoperative Management

Echocardiography monitors cardiac function and hemodynamics during surgery, allowing the anaesthesiologist to make timely anaesthesia management and fluid therapy decisions.

Real-Time Cardiac Function Assessment: In the operating room, the assessment of systolic and diastolic function is done continuously to identify early myocardial stress or inadequate fluid resuscitation. These parameters thus guide therapy in optimizing cardiac performance while preventing fluid overload [15].

Assessment of Hemodynamic Changes: It also aids in monitoring hemodynamic changes by assessing intravascular volume and fluid status via cardiac chamber sizes and fluid collections. Data from such an assessment is handy in guiding fluid resuscitation and diuretic therapy and influences anaesthesia and pharmacologic interventions [16].

Postoperative Management

Postoperative management has further critical roles in monitoring cardiac function and managing complications during the postoperative period by adjusting care for changes evolving with the patient, thereby optimizing recovery and outcome.

Monitoring Cardiac Function: Early postoperative echocardiography evaluates cardiac function and guides further management. Serial EF and diastolic function changes identify myocardial ischemia or fluid imbalance and guide medication and fluid management decisions [17].

Identification of Complications and Their Management: Echocardiography in the early phase of postoperative care identifies myocardial ischemia, pericardial effusion, or valve malfunction. Its early identification allows the institution of early measures against such complications, thus avoiding deterioration in the patient's condition. Finally, the effects of fluid balance and medication need to be assessed appropriately in postoperative care. The measurements obtained by echocardiography of cardiac chamber size and wall thickness enable guiding fluid therapy adjustments, and the effects of drugs such as vasopressors and inotropes are monitored [18].

Training and competency in anaesthesiology use of echocardiography

There is a need for training and certification of anaesthesiologists in echocardiography because cardiac performance assessment during surgery is complex and vital. Echocardiography will provide all information related to cardiac function, fluid status, and hemodynamics that help guide anaesthetic management while ensuring the safety of all patients. Image interpretation with accuracy is a requirement of decision-making in fluid resuscitation, inotropic support, and other areas of managing cardiac patients needing surgical intervention. Misinterpreted echocardiography data can result in inappropriate clinical decisions and thus negatively impact patient outcomes. Inadequate assessment of cardiac function or volume status can be linked to improper fluid management or administration of inappropriate drugs. It is a good reason to provide every anaesthesiologist with formal echocardiography training regarding the technical issues of image acquisition and an understanding of the clinical implications of the findings. Certification reassures patients, colleagues, and healthcare institutions that the practitioner has satisfied predefined criteria for skills and knowledge in echocardiography. Certification also elevates anaesthesiologists' credibility and demands quality care with uniform and accurate echocardiographic assessment [19]. Several societies provide guidelines and certification processes that help maintain standards of practice for anaesthesiologists who perform echocardiography. These include the American Society of Echocardiography (ASE) and the National Board of Echocardiography (NBE). The ASE provides guiding principles regarding echocardiography and does so with thresholds established for certification. Much focused on cardiovascular imaging, its standards apply to anaesthesiologists utilizing echocardiography in perioperative practice. ASE provides an organized certification by a written and practical examination. There is particular interest placed on the technique of TEE. The guidelines of the ASE class require acquaintance with echocardiographic techniques and image interpretation. They also incorporate those findings into patient management. For certification, a candidate must be competent in performing and interpreting echocardiographic studies, including TEE, and meet eligibility criteria for educational and professional experience [20].

The NBE certifies various echocardiographic modalities relevant to anaesthesiology. It requires knowledge and skills-based, all-inclusive examination for certification. The advanced cardiac echocardiography certification by NBE benefits anaesthesiologists in handling relatively more advanced cardiac imaging encountered in the perioperative environment. Applicants through the NBE certification process must be competent in echocardiographic techniques and their application in clinical practice. That means anaesthesiologists should be skilled in performing echocardiographic studies and their interpretation within perioperative care. The rigorous standards set by NBE certification have long been considered exemplary in excellence in echocardiography [21]. Besides ASE and NBE, societies and other bodies provide training programs and similarly give certifications. For example, the Society of Cardiovascular Anesthesiologists (SCA) has guidelines on the performance of echocardiography by anaesthesiologists, addressing best practices, training requirements, and competency assessment associated with the perioperative environment [20]. Through continuing medical education (CME) programs, anaesthesiologists can gain knowledge of new echocardiographic techniques and have updated information regarding emerging technologies and changes to clinical guidelines. CME activities include workshops, seminars, online courses, and conferences. CME shall enable anaesthesiologists to acquire knowledge and technical expertise through such processes to keep pace with the development of echocardiography.

Hands-on Practice and Simulation

Furthermore, the same practical exposure to echocardiography is also required to maintain competence in its practice. The actual performance helps an anaesthesiologist develop his techniques and garner experience in image acquisition and interpretation. Simulation training enables the practice of echocardiographic techniques and the exercise of associated decision-making; hence, it keeps the anaesthesiologist proficient in performing echocardiography during surgery and prepares the anaesthesiologist for complex cases [22].

Challenges and limitations of echocardiography in anaesthesia

Echocardiography use is fraught with challenges and limitations that bear on the accuracy and efficiency of echocardiographic evaluation and, thus, patient outcomes. These are elaborated upon in the current section and include operator dependency, patient factors, the invasive nature of TEE, and technological and interpretational shortcomings. Undeniably, one of the toughest challenges anaesthesia faces in echocardiography is strong operator dependence. Better image quality and accuracy regarding the echocardiographic images and a proper understanding of their meanings are definite skill and experience-related issues. In other words, steep learning curves with multiple imaging techniques, TTE, and TEE demand extensive training and experience in technical complexity and image acquisition, as well as the acquisition of precise knowledge of anatomy [23]. Patient-related factors are another source of error that grossly influences the effectiveness of echocardiography. It includes physiological and pathological conditions in a patient, which may alter the echocardiographic image quality, leading to an erroneous assessment. These limitations are often exaggerated in subgroups of patients such as the obese, those with lung disease, or those with anatomic abnormalities. Excess body fat in such patients can attenuate the ultrasound waves to produce precise imaging of the cardiac structures, which could be highly difficult, especially with TTE. The windows in severe obesity will be poor, the clarity of the image will be low, and there could be complications in assessing the cardiac status [24]. Conditions such as chronic obstructive pulmonary disease or interstitial lung disease produce more air in the chest cavity, interfering with ultrasound wave conduction and decreasing the quality of images. Severe pulmonary pathology may change hemodynamics and thus confound the interpretation of echocardiographic findings. Anatomical variations present an echocardiographic diagnosis as challenging because of the variations or anomalies against the normal expected anatomy. These anomalies require alterations to the technique to accommodate anatomic variations for optimum study [25]. While TEE gives exquisite images of the heart, especially in cardiac surgery, it is relatively invasive and associated with certain limitations and risks. TEE also requires the introduction of an endoscope with an ultrasound transducer into oesophagal missed views, making it more invasive compared to transthoracic approaches. Accordingly, sedation or even anaesthesia is needed to perform the technique. Therefore, the procedure becomes more complicated, and the risks hugely increase, especially in those patients who have many comorbid conditions.

One may consider the possibility of the invasiveness of TEE causing oesophagal trauma, bleeding, or perforation, which is relatively low. Also, the very invasive nature of the process may not be tolerated well by the patient, and it can be anxiety-provoking or uncomfortable. Another limitation is that TEE cannot visualise some thoracic structures well. It would again limit the completeness of the cardiac evaluation. Anaesthesiologists must appreciate these limits and bring other imaging modalities or more clinical assessment into the picture at appropriately indicated points [26]. In this day and age, image resolution can be increased with modern technology. Still, many are of such shallow quality that they lack the detail necessary for properly evaluating cardiac structure and function, thus affecting clinical decisions. Echocardiographic images are also often degraded by artefacts. In particular, the patient's movement causes motion artefacts, which permit compromised echocardiographic visualization, while acoustic shadowing, for example, by bone or air-disturbs echocardiographic visualization and interpretation [27]. Interpretative variability images in echocardiography may have different interpretations from experienced operators, depending on the degree of cardiac pathology, the image quality, and the operator's expertise. In this regard, using echocardiographic findings is essential because they correlate.

Technology development in echocardiography: novelty and future directions

The progress of cardiac imaging in the last 20 years has made echocardiography more accurate, convenient, and efficient in providing better patient care and outcomes. A few emerging trends are opening new doors to applications of echocardiography. 3D echocardiography has continued evolving and developing beyond 2D imaging because it provides volumetric imaging for the detailed study of cardiac anatomy and pathology. 3D echocardiography did improve a great deal in the imaging of complex structures such as heart valves and chambers. It changed congenital heart defect evaluation, the assessment of valvular lesions, and interventional procedures. It also provides accurate measurements for procedural planning such as valve repair or replacement and complex heart modelling to understand ventricular strain and desynchrony in managing heart failure and other conditions [28]. AI technologies, especially machine learning algorithms, make intelligent, accurate, effective, and impactful echocardiographic imaging toward better interpretation. AI can automate image acquisition, processing, and interpretation, reducing operator dependency for improved consistency and precision. AI algorithms can identify cardiac structures and abnormalities, including valvular lesions and chamber enlargement, more precisely than ever before, with measurements needed to estimate the size and function of the heart automatically. Therefore, it becomes possible to incorporate other information from other imaging strategies and different resources regarding clinical history to improve the quality and completeness of an echocardiographic investigation [29].

Future trends and possible developments

Techniques from other imaging modalities, such as magnetic resonance imaging (MRI) and computed tomography (CT), can be combined with echocardiography to offer complementary details that help diagnose and manage cardiac diseases. Hybrid imaging approaches will exploit the strength of the modality to enhance diagnostic accuracy and decisions on therapy. For instance, when combined with CT angiography, echocardiography provides the optimal assessment of coronary artery disease and offers guiding principles for interventional procedures [30]. Echocardiography can be matched with genetic, molecular, and clinical information to provide personalized medicine and precision care for cardiovascular diseases. Under genomic evaluation and biomarkers, the integration of personalized echocardiographic examinations aids in the construction of high-quality forecasts of the progress of diseases, treatment responses, and inventions tailored to patients' needs [31]. The future of wearable and portable echocardiographic technologies will further emphasise point-of-care imaging, allowing patients to be monitored remotely. It can and should be easy, user-friendly devices that allow continuous monitoring of cardiac function, early detection of dysfunction, and timely interventions, especially in high-risk populations and those with chronic diseases [32]. Virtual and augmented reality technologies can enrich education and training on echocardiography methodologies, making them immersive and interactive. These techniques can simulate complex heart anatomy and pathology, significantly improving learning outcomes and developing skills among healthcare professionals [33].

Impact on clinical decision-making and patient outcome

Echocardiography has significant effects on clinical decision-making and the care of patients. The technique gives a real-time dynamic assessment of cardiac function, which is invaluable in tailoring anaesthesia management, especially in high-risk surgeries and those with complex cardiovascular conditions. The anaesthesiologist will be able to pick up and manage myocardial dysfunction, valvular abnormalities, and changes in volume status during surgery, thus optimizing interventions. It has opened new horizons of self-assuredness in anaesthesiologists about whether they can manage intraoperative hemodynamics. With real-time feedback regarding cardiac performance in patients, anaesthesiologists can precisely titrate vasoactive medications, fluids, and inotropic support and hold at least some associated risks of these interventions. Such real-time feedback manages patients more safely and yields better outcomes in surgeries with high hemodynamic variability. An early intraoperative session echocardiographic assessment of cardiac valves may lead to the optimal repair of the cardiac valve or its replacement and hence avoid postoperative complications later on. Intraoperative monitoring and detection of echocardiography-associated myocardial ischemia ensure quick intervention to minimise the events of adverse cardiac outcomes. It places echocardiography in the category of a modality that makes a considerable contribution toward patient safety and successful surgery. Anaesthesiologists are conversant that attaining and mastering echocardiographic prowess in this resourced field comes with professional fulfilment. Such advanced cardiac imaging ability integrated into practice opens new opportunities and contributes to professional satisfaction and augmentation. Many anaesthesiologists take pride in echocardiography, knowing their positive impact on patient care and clinical outcomes. Professional development is gained from continuous learning entailed by echocardiography both for staying updated on its rapidly evolving technology and the current guidelines [34].

## Conclusions

In conclusion, echocardiography has become an extremely important modality in anaesthesiology. Echocardiography has revolutionized monitoring and managing cardiac function intraoperatively. From its conceptual development using 2D and 3D basic imaging to the highly sophisticated techniques now under development, including work on integrating artificial intelligence, progress in the field has greatly empowered the anaesthesiologist to make interventions increasingly individualized and maximally beneficial to patients in real time. Echocardiography is important in perioperative patient care despite inherent challenges: operator dependence, learning curves, and many technological and patient limitations. Continuing professional development and technological advancement, especially in 3D and AI, will significantly enhance this role to be central in improving patient outcomes and surgical success. With the development, anaesthesiologists are compelled to grow with it to fully exploit echocardiography in delivering personalized and quality care.

## References

[1] Cheitlin MD, Alpert JS, Armstrong WF (1997). ACC/AHA guidelines for the clinical application of echocardiography: a report of the American College of Cardiology/American Heart Association Task Force on practice guidelines (Committee on Clinical Application of Echocardiography). Developed in collaboration with the American Society of Echocardiography. Circulation.

[2] Tank A, Hughey R, Ward RP, Nagele P, Rubin DS (2021). Evaluation of appropriate use of preoperative echocardiography before major abdominal surgery: a retrospective cohort study. Anesthesiology.

[3] Pellegrini JA, Mendes CL, Gottardo PC (2023). The use of bedside echocardiography in the care of critically ill patients - a joint consensus document of the Associação de Medicina Intensiva Brasileira, Associação Brasileira de Medicina de Emergência and Sociedade Brasileira de Medicina Hospitalar. Part 2 - Technical aspects. Crit Care Sci.

[4] Uppot RN (2018). Technical challenges of imaging & image-guided interventions in obese patients. Br J Radiol.

[5] Maleki M, Esmaeilzadeh M (2012). The evolutionary development of echocardiography. Iran J Med Sci.

[6] Grant MD, Mann RD, Kristenson SD (2021). Transthoracic echocardiography: beginner's guide with emphasis on blind spots as identified with CT and MRI. Radiographics.

[7] Puchalski MD, Lui GK, Miller-Hance WC (2019). Guidelines for performing a comprehensive transesophageal echocardiographic examination in children and all patients with congenital heart disease: recommendations from the American Society of Echocardiography. J Am Soc Echocardiogr.

[8] Steeds RP, Wheeler R, Bhattacharyya S (2019). Stress echocardiography in coronary artery disease: a practical guideline from the British Society of Echocardiography. Echo Res Pract.

[9] Schoenhagen P, Nissen S (2002). Understanding coronary artery disease: tomographic imaging with intravascular ultrasound. Heart.

[10] Wu VC, Takeuchi M (2017). Three-dimensional echocardiography: current status and real-life applications. Acta Cardiol Sin.

[11] Stewart MJ (2003). Contrast echocardiography. Heart.

[12] Subramani S, Tewari A (2014). Pre-operative echocardiography: evidence or experience based utilization in non-cardiac surgery. J Anaesthesiol Clin Pharmacol.

[13] Ryu T, Song SY (2017). Perioperative management of left ventricular diastolic dysfunction and heart failure: an anesthesiologist's perspective. Korean J Anesthesiol.

[14] Zoghbi WA, Jone PN, Chamsi-Pasha MA (2024). Guidelines for the evaluation of prosthetic valve function with cardiovascular imaging: a report from the American Society of Echocardiography developed in collaboration with the Society for Cardiovascular Magnetic Resonance and the Society of Cardiovascular Computed Tomography. J Am Soc Echocardiogr.

[15] Henein MY, Lindqvist P (2020). Diastolic function assessment by echocardiography: a practical manual for clinical use and future applications. Echocardiography.

[16] De Backer D, Aissaoui N, Cecconi M (2022). How can assessing hemodynamics help to assess volume status. Intensive Care Med.

[17] Chin AJ, Vetter JM, Seliem M, Jones AA, Andrews BA (1994). Role of early postoperative surface echocardiography in the pediatric cardiac intensive care unit. Chest.

[18] Pepi M, Muratori M, Barbier P (1994). Pericardial effusion after cardiac surgery: incidence, site, size, and haemodynamic consequences. Br Heart J.

[19] Pospishil L, Hoffmeister KJ, Neuburger PJ (2023). Special competency in echocardiographic guidance for structural heart disease interventions: cardiac anesthesiologists as interventional echocardiographers. J Cardiothorac Vasc Anesth.

[20] Hahn RT, Abraham T, Adams MS (2013). Guidelines for performing a comprehensive transesophageal echocardiographic examination: recommendations from the American Society of Echocardiography and the Society of Cardiovascular Anesthesiologists. J Am Soc Echocardiogr.

[21] Hahn RT, Mahmood F, Kodali S (2019). Core competencies in echocardiography for imaging structural heart disease interventions: an expert consensus statement. JACC Cardiovasc Imaging.

[22] Singham A, Paul A (2024). Simulation-based training for anaesthesiology residents: a boon. J Clin Diagn Res.

[23] Ali SI, Li Y, Adam M, Xie M (2019). Evaluation of left ventricular systolic function and mass in primary hypertensive patients by echocardiography. J Ultrasound Med.

[24] Ellenberger K, Jeyaprakash P, Sivapathan S (2022). The effect of obesity on echocardiographic image quality. Heart Lung Circ.

[25] Zanforlin A, Smargiassi A, Inchingolo R, Valente S, Ramazzina E (2015). Ultrasound in obstructive lung diseases: the effect of airway obstruction on diaphragm kinetics. A short pictorial essay. J Ultrasound.

[26] Mathur SK, Singh P (2009). Transoesophageal echocardiography related complications. Indian J Anaesth.

[27] Le HT, Hangiandreou N, Timmerman R, Rice MJ, Smith WB, Deitte L, Janelle GM (2016). Imaging artifacts in echocardiography. Anesth Analg.

[28] Lang RM, Mor-Avi V, Sugeng L, Nieman PS, Sahn DJ (2006). Three-dimensional echocardiography: the benefits of the additional dimension. J Am Coll Cardiol.

[29] Barry T, Farina JM, Chao CJ (2023). The role of artificial intelligence in echocardiography. J Imaging.

[30] Hussain S, Mubeen I, Ullah N (2022). Modern diagnostic imaging technique applications and risk factors in the medical field: a review. Biomed Res Int.

[31] Sethi Y, Patel N, Kaka N (2023). Precision medicine and the future of cardiovascular diseases: a clinically oriented comprehensive review. J Clin Med.

[32] Liao Y, Thompson C, Peterson S, Mandrola J, Beg MS (2019). The future of wearable technologies and remote monitoring in health care. Am Soc Clin Oncol Educ Book.

[33] Tene T, Vique López DF, Valverde Aguirre PE, Orna Puente LM, Vacacela Gomez C (2024). Virtual reality and augmented reality in medical education: an umbrella review. Front Digit Health.

[34] Durai Samy NK, Taksande K (2024). Revolutionizing cardiac anesthesia: a comprehensive review of contemporary approaches outside the operating room. Cureus.

